# Green synthesis of ZnO-TiO_2_/RGO nanocomposites using *Senna surattensis* extract: a novel approach for enhanced anticancer efficacy and biocompatibility

**DOI:** 10.1039/d4ra01634c

**Published:** 2024-05-22

**Authors:** ZabnAllah M. Alaizeri, Hisham A. Alhadlaq, Saad Aldawood, Naaser A. Y. Abduh

**Affiliations:** a Department of Physics and Astronomy, College of Science, King Saud University Riyadh 11451 Saudi Arabia zabn1434@gmail.com; b Department of Chemistry, College of Science, King Saud University Riyadh 11451 Saudi Arabia

## Abstract

The purpose of the present study is to enhance the anticancer and biocompatibility performance of TiO_2_ NPs, ZnO NPs, ZnO-TiO_2_ (NCs), and ZnO-TiO_2_/reduced graphene oxide (RGO) NCs against two types of human cancer (HCT116) and normal (HUVCE) cells. A novel procedure for synthesizing ZnO-TiO_2_/RGO NCs has been developed using *Senna surattensis* extract. The improved physicochemical properties of the obtained samples were investigated using different techniques such as XRD, TEM, SEM, XPS, FTIR, DLS and UV-visible spectroscopy. XRD results showed that the addition of ZnO and RGO sheets affects the crystal structure and phase of TiO_2_ NPs. SEM and TEM images displayed that the TiO_2_ NPs and ZnO NPs were small with uniform spherical morphology in the prepared ZnO-TiO_2_/RGO NCs. Besides, it is shown that ZnO-TiO_2_ NCs anchored onto the surface of RGO sheets with a particle size of 14.80 ± 0.5 nm. XPS data confirmed the surface chemical composition and oxidation states of ZnO-TiO_2_/RGO NCs. Functional groups of prepared NPs and NCs were determined using FTIR spectroscopy. DLS data confirmed that the addition of ZnO and RGO sheets improves the negative surface charge of the prepared pure TiO_2_ NPs (−22.51 mV), ZnO NPs (−18.27 mV), ZnO-TiO_2_ NCs (−30.20 mV), and ZnO-TiO_2_/RGO NCs (−33.77 mV). Optical analysis exhibited that the bandgap energies of TiO_2_ NPs (3.30 eV), ZnO NPs (3.33 eV), ZnO-TiO_2_ NCs (3.03 eV), and ZnO-TiO_2_/RGO NCs (2.78 eV) were further enhanced by adding ZnO NPs and RGO sheets. This indicates that the synthesized samples can be applied to cancer therapy and environmental remediation. The biological data demonstrated that the produced ZnO-TiO_2_/RGO NCs show a more cytotoxic effect on HCT116 cells compared to pure TiO_2_ NPs and ZnO-TiO_2_ NCs. On the other hand, these NCs displayed the lowest level of toxicity towards normal HUVCE cells. These results indicate that the ZnO-TiO_2_/RGO NCs have strong toxicity against HCT116 cells and are compatible with normal cells. Our results show that the plant extract enhanced the physicochemical properties of NPs and NCs compared with the traditional chemical methods for synthesis. This study could open new avenues for developing more effective and targeted cancer treatments.

## Abstract 2

The aim of the present study is to enhance the anticancer and biocompatibility performance of TiO_2_ and ZnO nanoparticles, and, ZnO-TiO_2_ and ZnO-TiO_2_/reduced graphene oxide nanocomposites, against two types of human cancer (HCT116) and normal (HUVCE) cells.

## Graphical abstract

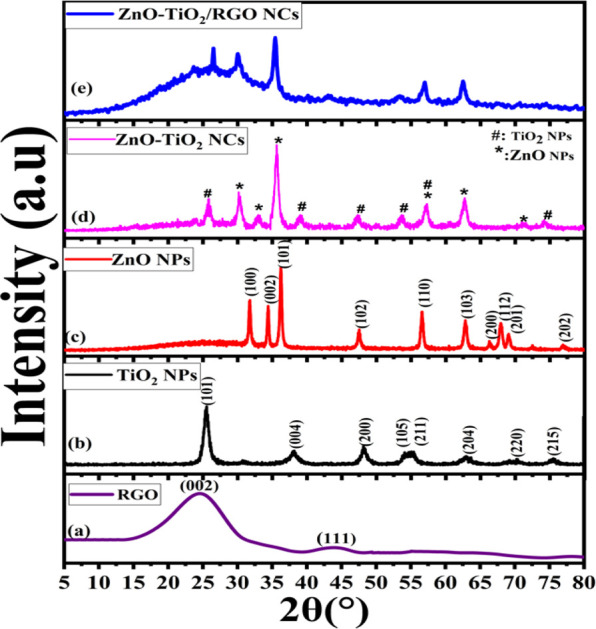

## 1. Introduction

Cancer is a major worldwide health issue, requiring the development of new and innovative treatment approaches.^1^ Currently, nanotechnology and nanomaterials science have attracted attention to address this challenge by providing approaches to improve the effectiveness and specificity of anticancer therapy.^2^ Among these nanomaterials, metal oxide nanoparticle-based nanocomposites (NCs) have attracted much interest owing to their unique properties and applications. Besides, they have been applied in a wide range of applications including catalysis, energy storage, and biomedical engineering.^3,4^ Many previous studies have reported the use of Ag_2_O/TiO_2_,^5^ WO_3_/ZnO NCs,^6^ SnO_2_/MgO NCs,^7^ In_2_O_3_/ZnO NCs^8^ in antibacterial, and anticancer applications due to their excellent properties. Additionally, the physicochemical properties of these NCs can be enhanced by the addition of another material (graphene oxide (GO), reduced graphene oxide (RGO), and polymers) by improved synthesis processes.

Different approaches have been explored for preparation and biomedical applications of different metal oxide NPs with reduced graphene oxide (RGO) to improve their physicochemical properties.^9,10^ For instance, fruit extract (*Phoenix dactylifera* L.) was used to synthesize Mo-ZnO/RGO NCs by Ahamed *et al.*^10^ The authors observed that the biocompatibility and anticancer performance were increased in green stabilized Mo-ZnO/RGO NCs compared with pure ZnO NPs. In another study, the TiO_2_/reduced graphene oxide NCs synthesized by the anodization method displayed excellent analytical capability in detecting MCF-7 cancer cells.^11^ Saravanan *et al.*^12^ used the thermal decomposition method to prepare ZnO/Ag/Mn_2_O_3_ NCs with enhanced antimicrobial activity.

To enhance the anticancer properties of these nanocomposites (NCs), several researchers have focused on combining two metal oxides with RGO as NCs in biomedical applications. For example, Ahamed *et al.*^13^ used ginger rhizome extract to prepare ZrO_2_-doped ZnO/rGO NCs, which exhibited far greater anticancer activity on lung (A549) and breast (MCF-7) cancer cells than pure ZnO NPs. Yao *et al.*^14^ synthesized SnO_2_/TiO_2_/RGO NCs using green hydrothermal methods with increased visible-light photocatalytic and antibacterial activity. Hossain *et al.*^15^ improved the antibacterial properties of TiO_2_-MWCNT NCs by doping with Fe or Ag, which reduced their bandgap energy. Ahamed *et al.*^16^ reported a green synthesis approach that employs garlic clove extract to produce SnO_2-_ZnO/RGO NCs with improved anticancer activity against breast (MCF-7) cancer cells.

The present work aimed to fabricate ZnO-TiO_2_/RGO NCs by novel green synthesis for their biological response. Prepared NCs were successfully characterized by different analytical techniques, such as XRD, TEM, SEM, XPS FTIR, DLS and UV-visible spectroscopy. The selective anticancer activity of the samples was assessed using an MTT assay. Biochemical data indicate that the ZnO-TiO_2_/RGO NCs had strong cytotoxicity against HTC116 cells and great biocompatibility with normal HUVEC cells.

## 2. Experimental section

### 2.1 Materials, reagents, and cells

Titanium butoxide (Ti(O-But)_4_), zinc nitrate hexahydrate (Zn(NO_3_)_2_·6H_2_O), sodium hydroxide (NaOH), reduced graphene oxide (RGO), MTT (3-[4,5-dimethylthiazol-2-yl]-2,5-diphenyl tetrazolium bromide), and dimethyl sulfoxide (DMSO) were purchased from Sigma-Aldrich, St. Louis, MO, USA. All reagents were of analytical grade and used without additional purification. The human colorectal cancer (HCT116) and human umbilical vein endothelial (HUVEC) normal cell lines were acquired from the American Type Culture Collection (ATTC) based in Manassas, WV, USA.

### 2.2 Preparation of *Senna surattensis* extract

*Senna surattensis* flowers were collected from the campus of King Saud University (KSU), Riyadh, KSA. Then, it was cleaned with deionized water, shade-dried for two weeks, and then mixer-ground. Hence, the shade drying process was less affected by the chemical composition and biological activity of plants compared with oven drying. After drying, 10 g of *Senna surattensis* flowers were added to 100 ml of deionized water to prepare the extract solution. For optimal extraction of plant water-soluble components, the mixture solution was heated to 60 °C under stirring for 10 min. Lastly, the prepared extract solution was cooled, filtered with filter paper, and kept at 4 °C for future use in the present work.

### 2.3 Synthesis of ZnO-TiO_2_ NCs and ZnO-TiO_2_/RGO NCs

**Scheme 1:**
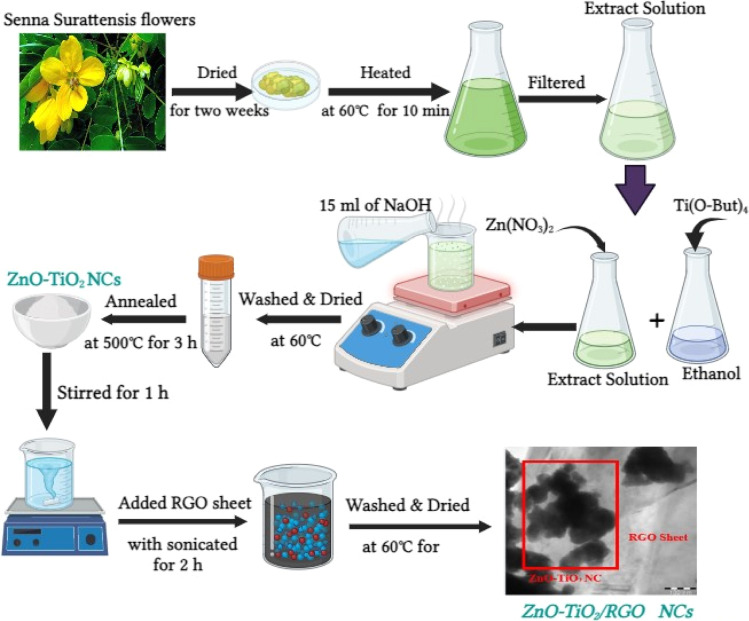
Synthesis procedures of ZnO-TiO_2_/RGO NCs.

4.37 g of Zn(NO_3_)_2_·6H_2_O was dispersed in 40 ml of extract aqueous solution in an Erlenmeyer flask. Next, 5 ml of Ti(O-But)_4_ was dissolved in 10 ml of ethanol dropwise. After that, 15 ml of NaOH solution (2 M) was added. Subsequently, the mixed solution was heated to 60 °C. Then, a separating process was used to remove the precipitate from the solution. Next, it was washed several times with water/ethanol at a volume ratio equal to (3/1). This precipitate was dried overnight in a 60 °C oven and annealed in a tube furnace at 500 °C for 3 h under atmospheric air. Pure ZnO NPs were prepared by the same route without Ti(O-But)_4_. Similarly, pure TiO_2_ NPs were synthesized without Zn(NO_3_)_2_·6H_2_O and NaOH solution. ZnO-TiO_2_/RGO NCs were also fabricated using a sonication process. Initially, 1 g of ZnO-TiO_2_ NCs and 10% of RGO sheets were further dispersed in 30 ml of deionized water under an ultra-sonicate wave at 80 kW for 2 h. Then, the mixture solution was dried in an oven at 60 °C overnight for 12 h to obtain ZnO-TiO_2_/RGO NCs as nanopowder. Pure TiO_2_ NPs, ZnO_2_ NPs, and ZnO-TiO_2_ NCs were synthesized without Zn(NO_3_) using RGO, Ti(O-But)_4_, and RGO sheets under the same conditions. The procedures for preparing ZnO-TiO_2_/RGO NCs are presented in Scheme 1.

### 2.4 Characterization techniques

X-ray diffraction (XRD) (PanAnalytic X'Pert Pro instrument from Malvern Instruments, UK) was applied to examine the crystalline structures and phases of synthesized NPs and NCs. Morphological and surface properties of prepared samples were further investigated through scanning electron microscopy (SEM) (JSM-7600F instrument from JEOL, Inc). X-ray photoelectron spectroscopy (XPS) (PerkinElmer PHI-5300 ESCA device in Boston, MA, USA) was used to confirm the chemical states and compositions of the produced samples. The functional groups of the obtained samples were recorded at a wavenumber range of 400 to 4000 cm^−1^ using Fourier transform infrared (FTIR) spectroscopy (PerkinElmer Paragon 500, USA). Dynamic light scattering (DLS) (Malvern Panalytical Ltd, located in Worcestershire, UK) was used to assess the surface charge of synthesized NPs and NCs. The optical properties of the obtained NPs and NCs were studied by UV-vis spectroscopy (Hitachi U-2600).

### 2.5 Anticancer and biocompatibility assessments by MTT bioassay

The HTC116 cancer cells and HUVECs normal cells were cultured in Dulbecco's Modified Eagle Medium (DMEM) with 10% Fetal Bovine Serum (FBS), 100 U mL^−1^ penicillin, and 10 mg L^−1^ streptomycin. For each cell line, 70–80% of cell growth was trypsinized in culture fluxes and plated on 96-well plates. After seeding 10^4^ cancer cells per well onto plates, they were allowed to adhere and develop overnight in 200 μL of culture media. Cells were cultured with new media with varied doses (3.125–200 μg ml^−1^) of prepared samples for 24 hours. The plates were incubated at 37 °C and 5% CO_2_ for 3 hours after adding 20 μL of MTT solution (5 μg ml^−1^) to each well with 100 μL of the medium. Post-incubation, the MTT solution was removed, and 100 μL of dimethyl sulfoxide (DMSO) was added to dissolve formazan crystals in each well. The plates were shaken for 20 minutes on a plate shaker to ensure solubility. The biocompatibility of prepared NPs was tested using the same methods.

### 2.6 Statistical analysis

This study used SPSS (SPSS Inc., Routledge, NY, USA) for all statistical analysis. Comparing mean values using Duncan's multiple range tests revealed significant differences (*p* < 0.05).

## 3. Results and discussion

### 3.1 XRD study

**Fig. 1:**
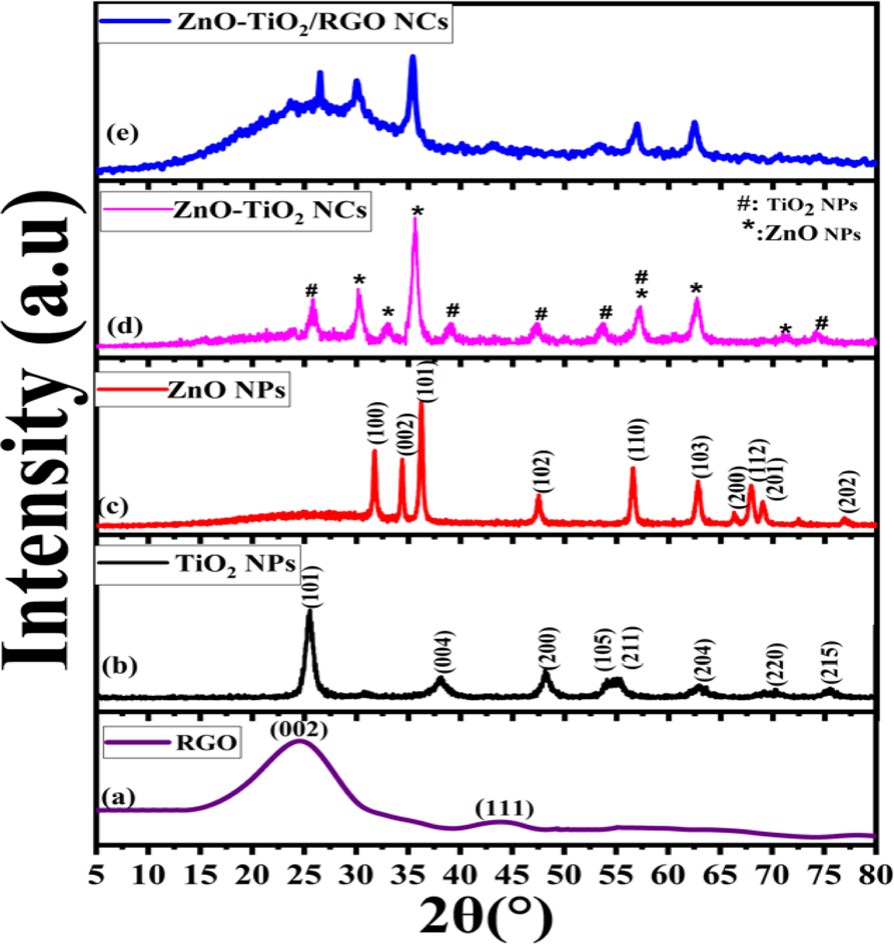
XRD spectra of RGO (a), TiO_2_ NPs (b), ZnO NPs (c), ZnO-TiO_2_ NCs (d), and ZnO-TiO_2_/RGO NCs (e).

XRD analysis provides significant insights into the crystal structure and composition of synthesized samples. XRD spectra of RGO (Fig. 1a) exhibit a strong peak at 2*θ* = 25°, corresponding to the graphene sheet interlayer spacing (002). Moreover, XRD spectra of RGO demonstrate a small peak at 2*θ* = 43°, indicating graphitic carbon (111) reflection.^17^ The XRD spectra of prepared TiO_2_ NPs, ZnO NPs, ZnO-TiO_2_ NCs, and ZnO-TiO_2_/RGO NCs are presented in Fig. 1b–e. As shown in Fig. 1b, the XRD peaks at 2*θ* values of 25.3°, 37.8°, 48.0°, 53.9°, 55.4°, 62.7°, 69.8°, and 75.6° indicate the crystallographic planes (101), (004), (200), (105), (204), (211), (220), and (215) of anatase TiO_2_. Similarly, XRD spectra of ZnO NPs (Fig. 1c) reveal peaks at 2*θ* values of 31.9°, 34.5°, 36.3°, 47.6°, 56.6°, 62.9°, 66.3°, 67.9°, 69.0°, and 77.2°, corresponding to wurtzite crystal structure planes (100), (002), (101), (102), (110), (103), (200), (112), (201), and (202), respectively. These results match with those from previous studies.^18,19^ Furthermore, the XRD spectra of ZnO-TiO_2_ NCs (Fig. 1d) show a mixture of the distinct peaks observed in TiO_2_ NPs and ZnO NPs, as reported in earlier study.^20^ This indicates that ZnO-TiO_2_ NCs were successfully prepared. Fig. 1e demonstrates the XRD spectra of synthesized ZnO-TiO_2_/RGO NCs, wherein reduced graphene oxide (RGO) affects the crystallite size and structure of prepared NCs, similar to previous studies.^21,22^ By applying the Scherrer equation, the average crystallite size (*D*) of peaks (100), (004), and (101) for TiO_2_ NPs, ZnO NPs, ZnO-TiO_2_ NCs, and ZnO-TiO_2_/RGO NCs were 8 ± 0.9 nm, 20 ± 0.2 nm, 14 ± 0.6 nm, and 11 ± 0.2 nm, respectively. Our XRD results suggest the crystal structures and phases with purity of prepared NPs and NCs, which were further examined and supported by FTIR data (Fig. 4).

### 3.2 TEM study

**Fig. 2:**
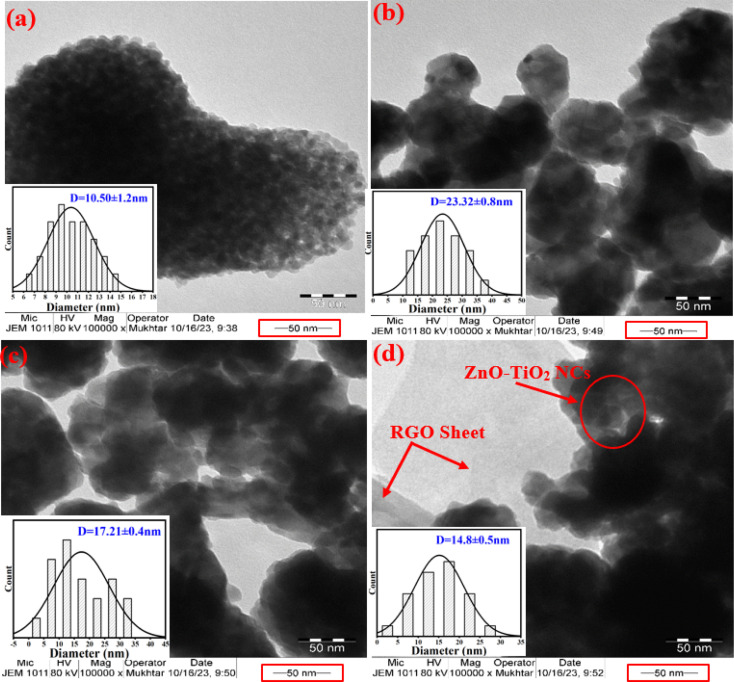
TEM characterization: TiO_2_ NPs (a), ZnO NPs (b), ZnO-TiO_2_ NCs (c), and ZnO-TiO_2_/RGO NCs (d).

The morphologies and particle sizes of synthesized NPs and NCs were imaged by the TEM technique, as illustrated in Fig. 2a–d. The TEM image (Fig. 2a) of TiO_2_ NPs was observed as small spherical particles with a uniform size distribution without any significant agglomeration, which is consistent with recent studies.^23,24^ Similarly, the TEM image of ZnO NPs exhibits a similar morphology to that of TiO_2_ NPs with aggregation, as reported in an earlier study.^25^ Nevertheless, the difference in these particle behaviors is due to several factors related to the specific properties of ZnO and TiO_2_ (surface area properties) and their interactions with the plant extract. As observed in Fig. 2c, the particles of ZnO NPs and TiO_2_ NPs are randomly distributed in ZnO-TiO_2_ NCs. Significantly, the TEM image of ZnO-TiO_2_/RGO NCs (Fig. 2d) confirmed that the ZnO-TiO_2_ NCs were integrated into the RGO sheets. In addition, the RGO sheets appear as thin, transparent layers, which are well-dispersed among the ZnO-TiO_2_ NCs. The histograms in Fig. 2a and b show the estimated diameters of TiO_2_ NPs, ZnO NPs, ZnO-TiO_2_ NCs, and ZnO-TiO_2_/RGO NCs were 10.50 ± 1.2 nm, 23.32 ± 0.8 nm, 17.21 ± 0.4 nm, and 14.80 ± 0.5 nm, respectively. These results from the TEM images were consistent with the data previously published.^26,27^

### 3.3 SEM study

**Fig. 3:**
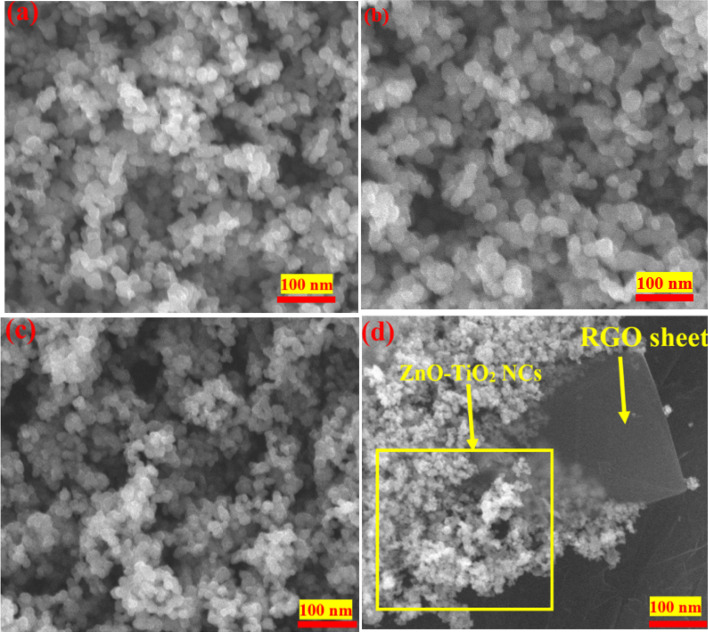
SEM images of TiO_2_ NPs (a), ZnO NPs (b), ZnO-TiO_2_ NCs (c), and ZnO-TiO_2_/RGO NCs (d).

Fig. 3a–d displays the SEM images of TiO_2_ NPs, ZnO-TiO_2_ NCs, and ZnO-TiO_2_/RGO NCs. The SEM image of TiO_2_ NPs (Fig. 3a) displays that the particles of TiO_2_ NPs were uniform with a smooth surface, matching with earlier studies.^28–30^ Thus, the particle size of ZnO NPs (Fig. 3b) was slightly higher, exhibiting the same shape and homogeneous distribution as reported in these investigations.^31,32^ In Fig. 3c, the SEM image of ZnO-TiO_2_ NCs exhibits fewer agglomeration particles with increased particle size due to combined TiO_2_ NPs and ZnO NPs.^33^ Compared to TiO_2_, the morphology of prepared ZnO-TiO_2_ NCs was similar to ZnO NPs. As seen in the SEM image (Fig. 3d), the particle size of prepared ZnO-TiO_2_ NCs was decreased after attaching RGO sheets, supporting TEM observations (Fig. 2d) and as reported in a previous study.^34^ This phenomenon indicates that the ZnO-TiO_2_/RGO NCs were successfully prepared. One the other hand, the addition of RGO sheets leads to a change in the morphology of prepared TiO_2_ NPs, enhancing the photocatalytic activity of obtained NPs and NCs. SEM images revealed that the surface morphology of ZnO-TiO_2_/RGO NCs was enhanced due to the reduction in the particle size of these NCs.

### 3.4 XPS analysis

**Fig. 4:**
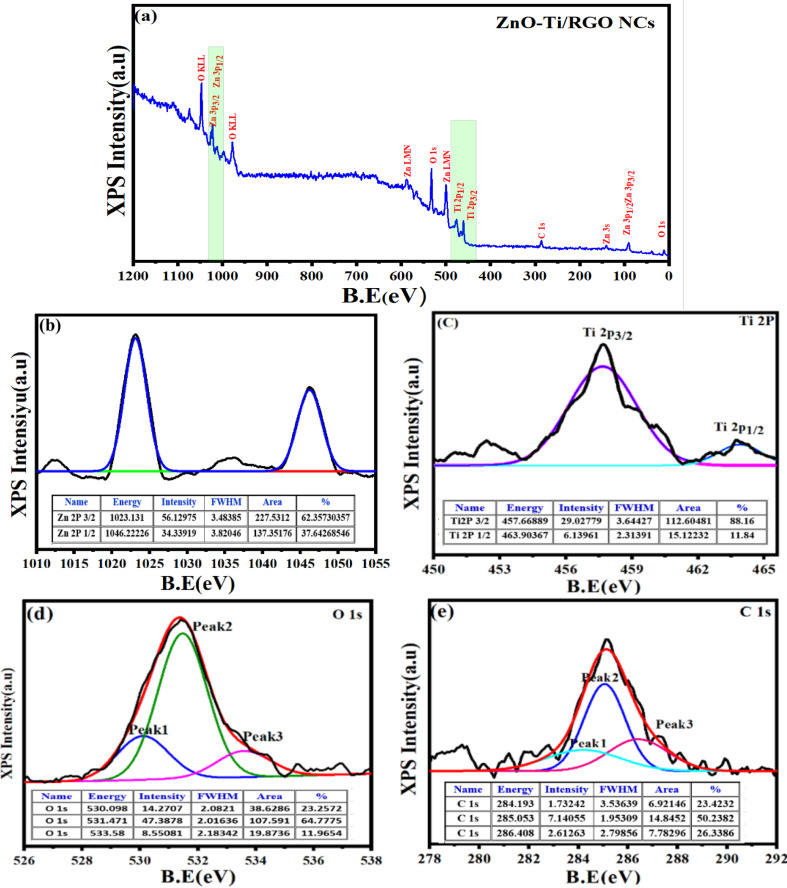
Full scan of XPS spectra of ZnO-TiO_2_/RGO NCs (a), XPS spectra of Zn 2p (b), XPS spectra of Ti 2p (c), XPS spectra of O 1s (d), and XPS spectra of C 1s (e).

The use of XPS spectroscopy is important in order to investigate the quantitative surface chemical composition and oxidation states of the prepared samples of ZnO-TiO_2_/RGO NCs, as presented in Fig. 4a–e. The presence of Zn 2p, Ti 2p, O 1s, and C 1s on the surface of ZnO-TiO_2_/RGO NCs was clearly observed in Fig. 4a. Additionally, the high-resolution XPS spectra of these elements are shown in Fig. 4b–e. The atomic concentration of each element present on the surfaces of ZnO-TiO_2_/RGO NCs was calculated using the following formula.^35^1
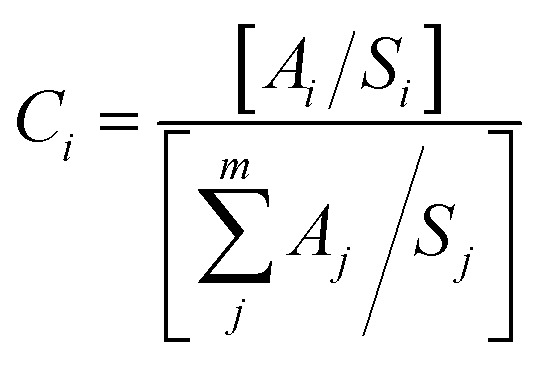
*A*_*j*_ represents the area of the photoelectron peak for the element “*i*”, *S*_*i*_ is the sensitivity factor for that element, and *m* is the total number of elements. The atomic concentrations of Zn, Ti, O, and C are presented in Table 1.

**Table 1:** Surface Composition Analysis of ZnO-TiO_2_/RGO NCs

Elements		Area/S	Atomic (%)
C	C 1s	118.59	21.64
O	C 1s	251.14	45.83
Zn	Zn 2p_1/2_	61.07	15.63
	Zn 2p_3/2_	24.58	
Ti	Ti 2p_1/2_	84.418	16.78
	Ti 2p_3/2_	7.56	

As shown in Fig. 4b, the XPS spectra of Zn 2p have two peaks at the binding energy of 1023.13 eV and 1046.22 eV for Zn 2p_3/2_ and Zn 2p_1/2_, respectively.^36^ Similarly, the two peaks of Ti 2p (Fig. 4c) were produced by spin-orbital coupling with binding energies of 457.66 eV and 463.90 eV for the Ti 2p_3/2_ and Ti 2p_1/2_ orbital states.^37^ The O 1s XPS spectra (Fig. 4d) showed three distinct peaks at 530.09 eV, 531.47 eV, and 533.58 eV.^38^ The peak at 531.47 eV is ascribed to the oxygen lattice in TiO_2_, whereas the peak at 530.09 eV and 533.58 eV were associated with surface defects, the O–H group bonded to Ti^4+^ covalently, and oxygen vacancy.^39^Fig. 4e displays the XPS spectra of C 1s, which have three peaks at 284.19 eV, 285.05 eV, and 286.40 eV.^40^ The presented XPS results agreed with earlier studies.^41,42^

### 3.5 FTIR study

**Fig. 5:**
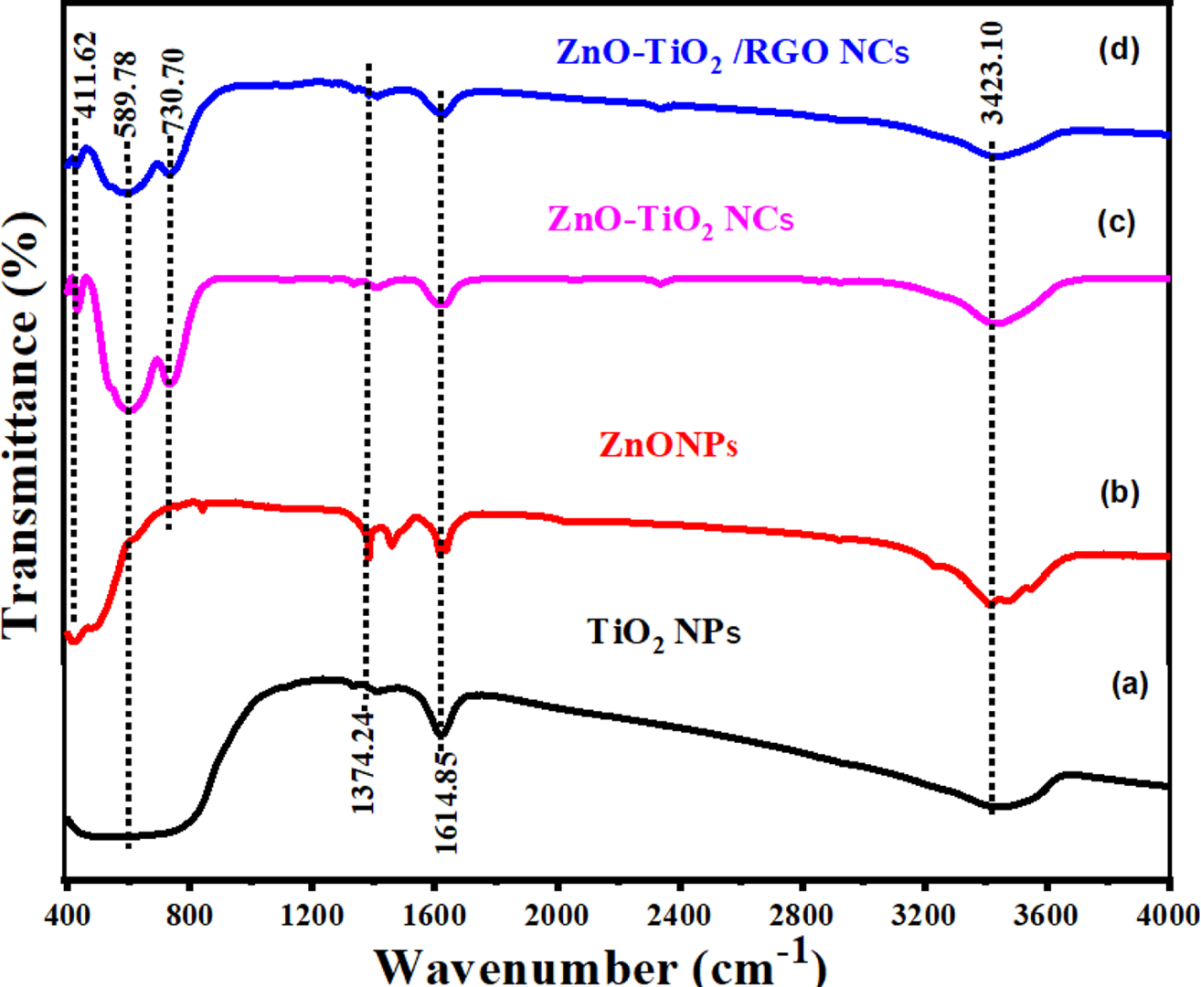
FTIR spectra of TiO_2_ NPs (a), ZnO NPs (b), ZnO-TiO_2_ NCs (c), and ZnO-TiO_2_/RGO NCs (d).

The chemical bonding and functional groups present in the prepared NPs and NCs were determined *via* FTIR technique, as depicted in the FTIR spectra (Fig. 5). As observed, the bands of synthesized samples at 411.62 cm^−1^ and 589.78 cm^−1^ were related to Zn–O and Ti–O bands, respectively.^33,43,44^ The peak observed at 730.70 cm^−1^ corresponds to the bending vibrations of the C=C bonds in both the prepared ZnO-TiO_2_ NCs and ZnO-TiO_2_/RGO NCs. The peaks detected at 1374.24 cm^−1^ and 1614.85 cm^−1^ were assigned to the stretching vibrations of C–O and C–H bonds, respectively, in agreement with an earlier study.^45,46^ Furthermore, the stretching vibrations of H–O groups were associated with the band at 3423.10 cm^−^,^1^ as reported in the previous study.^47^

### 3.6 UV-vis Measurements

**Fig. 6:**
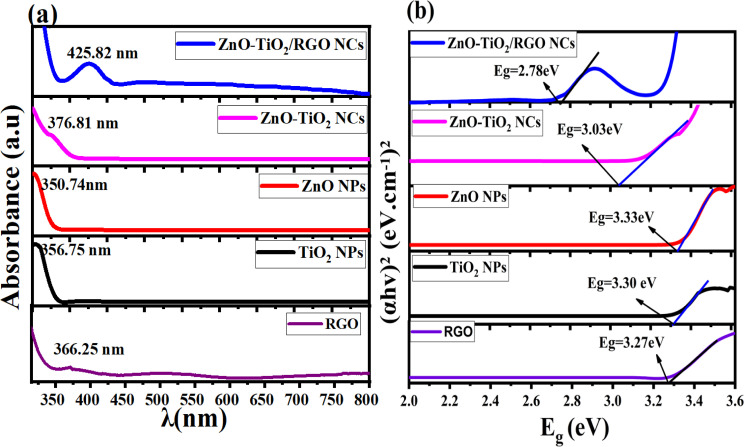
UV spectra of RGO, TiO_2_ NPs, ZnO NPs, ZnO-TiO_2_ NCs, and ZnO-TiO_2_/RGO NCs (a), and the optical bandgap energy of prepared samples (b).

Fig. 6a and b depict the UV-vis spectra and the optical bandgap energy of prepared samples, respectively. It can be observed in Fig. 6a that the absorption peaks exhibited in the UV and visible region are RGO (366.25 nm), TiO_2_ NPs (356.75 nm), ZnO NPs (350.74 nm), ZnO-TiO_2_ NCs (376.81 nm), and ZnO-TiO_2_/RGO NCs (425.82 nm), respectively, as reported in many studies.^26,27,48^ The absorption peaks have shifted toward the absorption edge, which was determined by UV absorbance. This shift indicates that there are changes in the band energy of synthesized NPs and NCs. The bandgap energy of these samples was further estimated by the Tauc eqn (2).^30^2
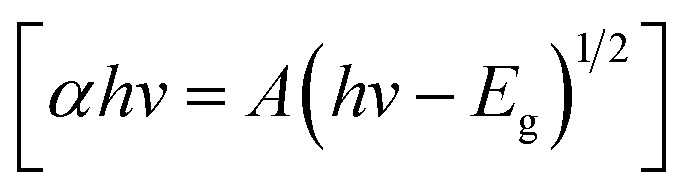


*A* is a constant, *E*_g_ is the bandgap energy of the sample, and 1/2 signifies a direct allowed transition. The calculated bandgap values (Fig. 6b) for RGO, TiO_2_ NPs, ZnO NPs, ZnO-TiO_2_ NCs, and ZnO-TiO_2_/RGO NCs were 2.27 eV, 3.30 eV, 3.33 eV, 3.03 eV and 2.87 eV, respectively. These values indicate that the bandgap energy (*E*_g_) of TiO_2_ NPs was tailored after the addition of ZnO and RGO sheets due to the efficient charge transport and the creation of oxygen vacancies.^43^ The UV findings indicate that the produced NCs may be used in photocatalytic and biological applications because of the increased absorption edge efficiency and reduced bandgap energy.

### 3.7 Surface charge analysis

**Fig. 7:**
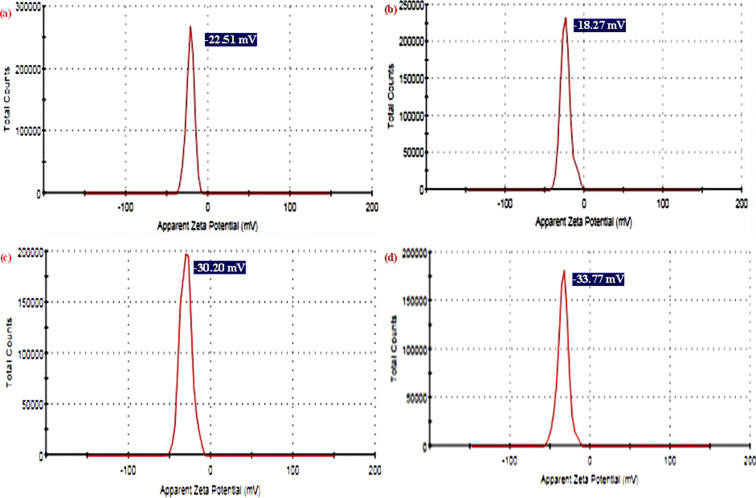
Zeta potential analysis of TiO_2_ NPs (a), ZnO NPs (b), ZnO-TiO_2_ NCs (c), and ZnO-TiO_2_/RGO NCs (d).

The dynamic light scattering (DLS) analysis was employed to measure the surface charge in the culture medium for synthesized NPs and NCs. As shown in Fig. 7a–d, the zeta potential values of pure TiO_2_ NPs, pure ZnO NPs, ZnO-TiO_2_ NCs, and ZnO-TiO_2_/RGO NCs were −22.51 mV, −18.20 mV, −30.2 mV, and −33.7 mV, respectively, as reported in this study.^49^ After the addition of ZnO NPs and RGO sheets, the negative zeta potential of TiO_2_ NPs was increased due to ZnO NPs and RGO sheets having a high surface charge.^50^ These negative charges indicated that the surface charge of NCs plays a role in determining their behavior in their interaction with cell membranes through electrostatic repulsion. Surface charge analysis confirmed that the negative zeta potential was essential for enhanced stability.^28,51^

### 3.8 Biological studies

#### 3.8.1 Cytotoxicity study

**Fig. 8:**
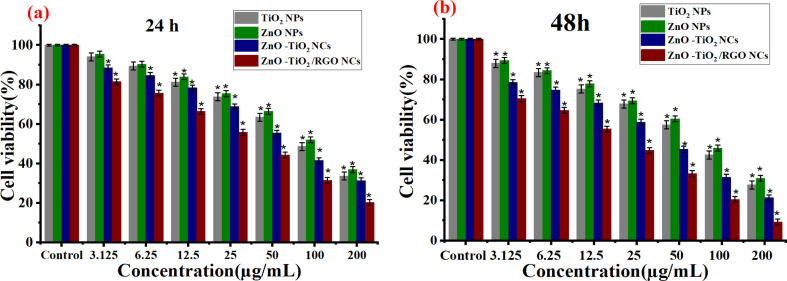
Cytotoxicity results of TiO_2_ NPs, ZnO NPs, ZnO-TiO_2_ NCs, and ZnO-TiO_2_/RGO NCs against HCT116 cells using MTT assay after 24 h (a) and 48 (b) exposure.

Metal oxide NPs and nanocomposites (NCs) have been applied in cancer therapy, as reported in many studies.^29,33,52–55^ In the present work, the anticancer activities of synthesized NPs at different concentrations (3.125–200 μg ml^−1^) against HTC 116 cells and NCs were assessed using MTT assay in the dark after 24 h and 48 h of exposure, respectively. As shown in Fig. 8a and b, the prepared TiO_2_ NPs exhibited high toxicity at high concentrations after 24 h and 48 h compared with the control group. Conversely, ZnO NPs displayed minimal cytotoxicity toward HCT116 cells after 24 h and 48 h of exposure (Fig. 8a and b), as shown in the previous study.^56^ It can be seen in Fig. 8a and b that the cell viability was highly decreased for ZnO-TiO_2_ NC due to the addition of the ZnO sheet due to their increasing surface charges. Similarly, the incorporation of RGO into ZnO-TiO_2_ NCs induces the highest cytotoxicity against HCT116 cancer cells at high concentrations (25, 50, 100, and 200 μg ml^−1^). It can be observed in Fig. 8a and b that the cell viability of HCT116 cancer cells was decreased with increasing exposure time due to prolonged exposure or the accumulation of reactive oxygen species. At a high concentration of 200 μg ml^−1^ at 48 h, the cell viability of HCT116 cancer cells of pure TiO_2_ NPs, pure ZnO NPs, ZnO-TiO_2_ NCs, and ZnO-TiO_2_/RGO NCs were 28.2%, 30.9%, 21.1%, 9.1%, respectively. The IC_50_ values of prepared NPs and NCs are presented in Table 2. Our results showed that the addition of ZnO NPs and RGO sheets plays a role in the high cytotoxicity of TiO_2_ NPs.

**Table 2:** IC_50_ values of prepared samples for HCT116 cells

NPs and NCs used	HCT116 cell lines	
IC_50_ (μg mL^−1^ ± SD)		
	24 h	48 h
TiO_2_ NPs	123.38 ± 0.9	105.02 ± 0.8
ZnO NPs	133.17 ± 0.4	113.79 ± 0.2
ZnO-TiO_2_ NCs	108.80 ± 0.7	79.44 ± 0.5
ZnO-TiO_2_/RGO NCs	77.82 ± 0.9	48.99 ± 0.3

#### 3.8.2 Biocompatibility evaluation

**Fig. 9:**
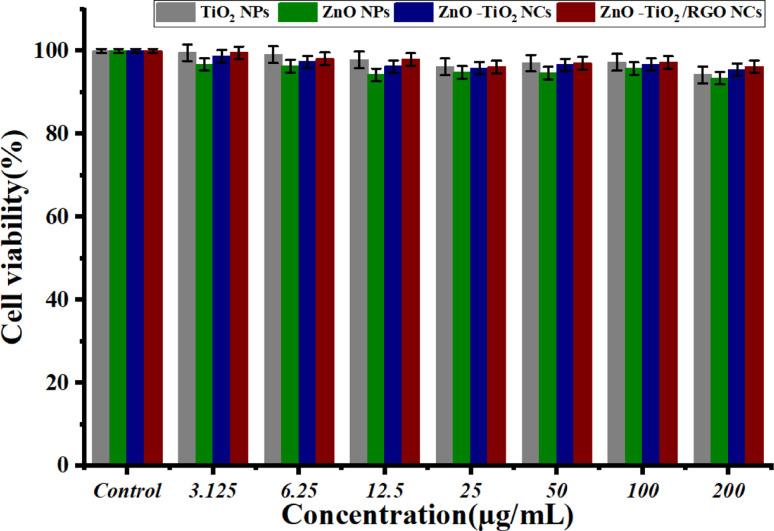
Biocompatibility results of TiO_2_ NPs, ZnO NPs, ZnO-TiO2 NCs, and ZnO-TiO_2_/RGO NCs against normal cells HUVCES cells by using MTT assay.

Several nanoparticles (NPs) and nanocomposites (NCs) have good biocompatibility with various normal human cells.^57–59^Fig. 9 shows the biocompatibility results of prepared NPs and NC against normal HUVCs. The findings showed that all NPs and NCs had excellent biocompatibility, as shown by the high cell viability of HUVEC cells. The findings indicate that the ZnO-TiO_2_/RGO NCs exhibit significant cytotoxicity against cancer cells and have great biocompatibility with normal cells. The results presented indicate that these samples are safe for normal cell lines and might be useful in biomedical applications. Due to its eco-friendliness, biocompatibility, and probable bioactivity, our plant extract enhances physicochemical NPs and NCs compared with traditional chemical methods for synthesis.

## 4. Conclusion

The present work proposed green synthesis and sonication routes to synthesize TiO_2_ NPs, pure ZnO NPs, ZnO-TiO_2_ NCs and ZnO-TiO_2_/RGO NCs. XRD, SEM with EDX, TEM, XPS, FTIR, UV-vis and DLS spectroscopy were applied to examine the physicochemical properties of the obtained NPs and NCs. XRD analysis confirms that the crystal structure of synthesized TiO_2_ is affected by adding ZnO and RGO sheets. TiO_2_ NPs, ZnO NPs, ZnO-TiO_2_ NCs, and ZnO-TiO_2_/RGO NCs displayed average crystallite sizes (*D*) of 8 ± 0.9, 20 ± 0.2, 14 ± 0.6, and 11 ± 0.2 nm, respectively. TEM and SEM images revealed that the addition of ZnO NPs and RGO plays a role in the particle size and morphology of TiO_2_ NPs. Furthermore, XPS analysis confirmed that prepared ZnO-TiO_2_/RGO NCs consisted of elements zinc (Zn), titanium (Ti), oxygen (O), and carbon (C) without impurities. FTIR revealed the chemical bonding and functional groups present in the synthesized NPs and NCs. UV-vis study showed that the bandgap energy of TiO_2_ decreased from 3.30 eV to 2.78 eV after ZnO and RGO sheets. DLS results showed that ZnO and RGO sheets increase the surface charge of pure TiO_2_ NPs (−22.51 mV), ZnO NPs (−18.27 mV), ZnO-TiO_2_ NCs (−30.20 mV), and ZnO-TiO_2_/RGO NCs (−33.77 mV). These results suggest that the prepared NPs and NCs can be used for photocatalytic and biomedical applications. MTT experiments were performed to assess the cytotoxicity and biocompatibility of synthesized NPs and NCs against HTC116 cancer and normal HUVEC cells for 24 h and 48 h exposure. These results showed that the cell viability of HCT116 cancer cells reduced with time due to extended exposure or reactive oxygen species formation. Furthermore, the ZnO-TiO_2_/RGO NCs exhibited the maximum cytotoxicity, suggesting cancer treatment potential. Also, the biocompatibility data on normal HUVEC cells showed that all synthesized NPs and NCs are safe for cancer therapy applications due to their low-toxic properties. Additionally, this study shows that the plant extract provides natural reducing and capping properties for NPs and NCs due to its eco-friendliness, biocompatibility, and possible bioactivity. This study suggests that cytotoxic effects and potential applications of these samples could be further investigated using *in vivo* models.

## Data availability

The data in this work may be obtained by contacting the corresponding author.

## Author contributions

Conceptualization was done by Z. M. A.; investigation and methods were carried out by Z. M. A., H. A. A., and S. A. Original draft preparation was done by Z. M. A. Review and editing were conducted by Z. M. A., H. A. A., S. A., and N. A. Y. All authors have reviewed and approved the final version of the text for publication.

## Conflicts of interest

This work is original research and has not been submitted for publication anywhere.

## Supplementary Material
